# Characterization of *FLOWERING LOCUS T1* (*FT1*) Gene in *Brachypodium* and Wheat

**DOI:** 10.1371/journal.pone.0094171

**Published:** 2014-04-09

**Authors:** Bo Lv, Rebecca Nitcher, Xiuli Han, Shuyun Wang, Fei Ni, Kun Li, Stephen Pearce, Jiajie Wu, Jorge Dubcovsky, Daolin Fu

**Affiliations:** 1 State Key Laboratory of Crop Biology, Shandong Key Laboratory of Crop Biology, Shandong Agricultural University, Tai’an, Shandong, China; 2 Department of Plant Sciences, University of California Davis, Davis, California, United States of America; 3 Howard Hughes Medical Institute, Chevy Chase, Maryland, United States of America; Institute for Sustainable Agriculture (IAS-CSIC), Spain

## Abstract

The phase transition from vegetative to reproductive growth is a critical event in the life cycle of flowering plants. *FLOWERING LOCUS T* (*FT*) plays a central role in the regulation of this transition by integrating signals from multiple flowering pathways in the leaves and transmitting them to the shoot apical meristem. In this study, we characterized *FT* homologs in the temperate grasses *Brachypodium distachyon* and polyploid wheat using transgenic and mutant approaches. Downregulation of *FT1* by RNAi was associated with a significant downregulation of the *FT-*like genes *FT2* and *FT4* in *Brachypodium* and *FT2* and *FT5* in wheat. In a transgenic wheat line carrying a highly-expressed *FT1* allele, *FT2* and *FT3* were upregulated under both long and short days. Overexpression of *FT1* caused extremely early flowering during shoot regeneration in both *Brachypodium* and hexaploid wheat, and resulted in insufficient vegetative tissue to support the production of viable seeds. Downregulation of *FT1* transcripts by RNA interference (RNAi) resulted in non-flowering *Brachypodium* plants and late flowering plants (2–4 weeks delay) in wheat. A similar delay in heading time was observed in tetraploid wheat plants carrying mutations for both *FT-A1* and *FT-B1*. Plants homozygous only for mutations in *FT-B1* flowered later than plants homozygous only for mutations in *FT-A1*, which corresponded with higher transcript levels of *FT-B1* relative to *FT-A1* in the early stages of development. Taken together, our data indicate that *FT1* plays a critical role in the regulation of flowering in *Brachypodium* and wheat, and that this role is associated with the simultaneous regulation of other *FT-*like genes. The differential effects of mutations in *FT-A1* and *FT-B1* on wheat heading time suggest that different allelic combinations of *FT1* homoeologs could be used to adjust wheat heading time to improve adaptation to changing environments.

## Introduction

The optimization of flowering and seed production is critical for plant survival and, in seed crops, to maximize grain yields. This is particularly important for cereal crops, which contribute significantly to global food production. However, the regulatory gene network controlling flowering is complex and its manipulation requires a precise understanding of the roles of different components and their interactions. In the temperate grasses, which include wheat, barley and the model species *Brachypodium distachyon*, vernalization (long exposures to cold temperatures) and photoperiod (variation in day length) are the main seasonal signals regulating flowering time. Over the last two decades, substantial progress has been made toward our understanding of the genes involved in these two pathways [1]–[3].

Vernalization requirement is conferred by the genes *VERNALIZATION1* (*VRN1*) [4], [5], *VRN2* [6], *VRN3* [7], and *VRN4* [8], [9]. Among these four genes, only the first three have been cloned and characterized to date. *VRN1* is a MADS-box meristem identity gene homologous to Arabidopsis *AP1*, which is upregulated during vernalization and promotes flowering in the spring [4]. *VRN2* is a floral repressor that encodes a protein containing a zinc finger motif and a CCT domain (CONSTANS, CONSTANS-LIKE, and TIMING OF CAB1-1) [6], and is downregulated by vernalization and short days (SD) [10], [11]. The *VRN3* gene encodes a RAF kinase inhibitor-like protein with high similarity to Arabidopsis protein FLOWERING LOCUS T (FT) and is a flowering promoter [7]. This gene will be referred to hereafter as *FT1* to differentiate it from other *FT-*like genes present in the temperate grasses ([12]).

These three vernalization genes are interconnected by complex interactions [3]. VRN2 acts as a repressor of *FT1* in the leaves, preventing flowering in the fall. During the winter, *FT1* and *VRN2* transcripts are reduced to almost undetectable levels whereas *VRN1* transcripts increase proportionally to the duration of the cold period [7]. At the end of the winter, the presence of VRN1 represses *VRN2* facilitating the upregulation of *FT1* during the lengthening days of spring [13]. *FT1* expression then further upregulates *VRN1* to reinforcing this inductive pathway and result in an irreversible transition to flowering.

Under long days (LD), *FT1* is upregulated by the photoperiod gene *PHOTOPERIOD 1* (*PPD1*), which is responsible for most of the natural variation in photoperiod sensitivity in wheat and barley [14]–[16]. The convergence of the photoperiod and vernalization pathways in the regulation of *FT1*, place this gene at the center of the gene network regulating flowering time in the temperate cereals [3].

In addition to its role in the integration of flowering signals, the small proteins encoded by *FT* and *FT-*like genes are essential for the transmission of environmental signals from the leaves to the shoot apical meristem, as demonstrated in Arabidopsis, rice and other species [17]–[20]. FT1 protein transport has yet to be demonstrated in the temperate cereals, but indirect evidence suggests that a similar mechanism exists in these species. FT1 is known to interact with the bZIP transcription factor FDL2, which binds to the promoter of *VRN1* [21]. Although *FT1* is not expressed in the wheat apex [22], a good correlation has been observed between the transcript levels of *FT1* in the leaves and of *VRN1* in the apex [21]. A significant correlation between flowering and *FT1* transcript levels in the leaves has also been observed in a sample of *Brachypodium* accessions [23].

Previous studies have shown that over-expression of *FT1* accelerates flowering in *Brachypodium* and wheat [24]–[26] but the effects of the downregulation of this gene in these species have not been reported before. In this study, we validate the *FT1* over-expression results and determine the effect of the downregulation of this gene by RNA interference (RNAi) in *Brachypodium* and wheat. We also use these transgenic lines to characterize the expression of the closest *FT-*like genes (*FT2* to *FT6*). Finally, we test the effect of mutations in *FT1* homoeologs in tetraploid wheat to determine their individual and combined contribution to heading time, and discuss their potential value for engineering wheat flowering time.

## Materials and Methods

The materials used in this study included the diploid *Brachypodium distachyon* (accession ‘Bd21-3′), diploid wheat *Triticum monococcum* (accession ‘G3116’), tetraploid wheat (*T. turgidum* ssp. *durum* cultivars ‘Kronos’ and ‘Langdon’), hexaploid wheat (*T. aestivum* cultivars ‘Bobwhite’ and ‘Jagger’), and a transgenic Jagger line carrying a highly expressed *FT-B1* allele from the variety Hope (referred to hereafter as *FT1*_HOPE_) driven by its native promoter [7].

*Brachypodium* plants were grown in a growth chamber at 25°C and LD photoperiod (16 h light/8 h dark, light intensity of 36 μmol m^−2^ s^−1^). Wild type Bobwhite plants used for transformation were grown in the field (University farm at Tai’an, Shandong, China). Transgenic plants for *FT1* silencing using RNA interference (*FT1*_RNAi_) were evaluated in greenhouses in China at 25°C and long days (16 h light/8 h dark, light intensity of 105 μmol m^−2^ s^−1^). Wheat mutants were evaluated in greenhouses in California under LD conditions (16 h light) with temperatures that oscillated between 21 and 23°C during the day and between 12 and 18°C during the night. *FT1*_HOPE_ overexpressing lines were evaluated in Conviron CMP3244 growth chambers (Conviron, Pembina, ND, USA) under LD (16 h light, 6∶00 a.m. –10∶00 p.m., 16°C) or SD (8 h light, 6∶00 a.m. –2∶00 p.m., 16°C).

### Plasmid Construction

To characterize the function of *FT1* genes, full-length *FT1* cDNAs were cloned from *Brachypodium* Bd21-3 (GT847109), *T. monococcum* G3116 (*FT-A^m^1*, DQ890163), and tetraploid wheat cultivar Langdon (*FT-B1*, DQ890164). The genomic region spanning the start to stop codons of the *FT-B1* gene was also cloned from Langdon (DQ890164).

**Figure 1:**
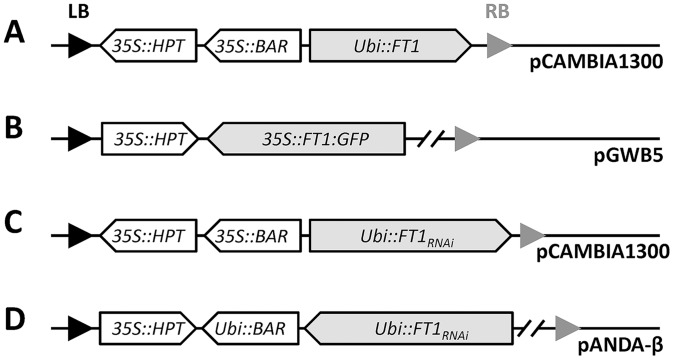
Schematic diagram of plasmids used in this study. (A) *Ubi::FT1* construct, (B) *35S::FT1:GFP* construct. Overexpression constructs were developed in binary vectors pCAMBIA1300 and pGWB5. For *Brachypodium FT1*, the cDNA was cloned in both vectors. For wheat *FT-B1*, both the coding and genomic regions were cloned in pCAMBIA1300. (C and D) *Ubi::FT1*_RNAi_ constructs. (C) The *Brachypodium FT1* RNAi trigger was cloned in the pCAMBIA1300-based vector. (D) The *FT1* RNAi trigger from *T. monococcum* was cloned in the pANDA-based vector. In both constructs, expression of the selected RNAi trigger was driven by the maize *Ubiquitin* promoter (*Ubi*). The black and gray triangles indicate the left (LB) and right (RB) border repeats respectively.

The *FT1* overexpression (*FT1*_OE_) constructs were developed in binary vectors pCAMBIA1300 and pGWB5 (Figure 1A–B) [27]. The pCAMBIA1300 vector was used for the overexpression of *FT1* cDNAs of Bd21-3 and Langdon, and the *FT1* genomic region of Langdon. The pGWB5 vector was used for the overexpression of a *Brachypodium FT1* cDNA fused to the green fluorescent protein (GFP).

For *FT1*_RNAi_, we selected fragments from *Brachypodium* Bd21-3 cDNA (position 79 to 447) and G3116 cDNA (position 62 to 367). The selected RNAi triggers did not share any stretch of more than 18 identical nucleotides with other *FT*-like genes, preventing co-silencing of other *FT-*like genes. The *Brachypodium FT1* RNAi trigger was cloned in the pCAMBIA1300-based vector (Figure 1C), and the wheat *FT1* RNAi trigger was cloned in the pANDA-based vector (Figure 1D) [28]. In both constructs, expression of the selected RNAi triggers was driven by the maize *Ubiquitin* promoter (*Ubi*).

### Generation of Transgenic Plants

Tissue culture and *Agrobacterium*-mediated transformation of *Brachypodium* was conducted as reported by Dr. Vogel and his colleagues [29]. Transgenic *Brachypodium* plants were established in soil in the growth chamber and in the greenhouse.

Protocols for the tissue culture and biolistic bombardment of wheat were adapted from a previous study [30]. Immature caryopses from *T. aestivum* cultivar Bobwhite were harvested two weeks after anthesis, sterilized with 70% (v/v) ethanol containing 0.05% (v/v) Tween 20 for 5 min, then with 20% (v/v) bleach supplemented with 0.05% (v/v) Tween 20 for 15 min, and washed 3–5 times using sterile distilled water. Immature embryos (ca. 1 mm long) were isolated from the sterilized caryopses, placed with the scutellum facing upward on the dissection media (MS base 4.3 g/L, maltose 40 g/L, thiamine-HCl 0.5 mg/L, L-asparagine 0.15 g/L, 2,4-D 2 mg/L, CuSO_4_ 0.78 mg/L, Phytagel 2.5 g/L, pH 5.8), and maintained for 4–6 days at 22–23°C in the dark. Immature embryos were then treated for four hours on the high osmoticum media (MS base 4.3 g/L, maltose 40 g/L, sucrose 171.15 g/L, thiamine-HCl 0.5 mg/L, L-asparagine 0.15 g/L, 2,4-D 2 mg/L, CuSO_4_ 0.78 mg/L, Phytagel 2.5 g/L, pH 5.8), and subjected to biolistic bombardment. Twenty hours after bombardment, immature embryos were transferred to recovery media (same as the dissection media), maintained for 2 weeks at 22–23°C in the dark. Embryo-derived calli were moved to the regeneration media (a dissection media supplemented with 0.1 mg/L 6-BA and 3 mg/L bialaphos) and maintained for two weeks in the growth chamber (22–23°C, 16 h light/8 h dark, light intensity of 25 μmol m^−2^ s^−1^). Regenerated shoots (2–3 cm) were transferred to the rooting media (a half-strength dissection media supplemented with 3 mg/L bialaphos), and maintained under the same environmental condition as for regeneration. Vigorous shoots with well-developed roots were established in soil in the greenhouse.

The biolistic bombardment was performed using the PDS-1000/He Particle Delivery System (Bio-Rad Laboratories, USA). To prepare three bombardments, 2.1 mg of microcarriers (Gold particles of 0.6 μm in diameter; Bio-Rad, USA) were measured into a 1.5 ml microcentrifuge tube, sterilized by mixing with 35 μl pure ethanol, recovered by spinning (12,000 rpm for 5 s) and removing the supernatant, rinsed in 200 μl ice-cold sterile distilled water, and collected by spinning and removing the supernatant. The pre-treated microcarriers were resuspended in 245 μl pre-chilled sterile water containing 20 μg plasmid DNA, and combined with another 250 μl pre-chilled CaCl_2_ (2.5 M). Where required, solutions in the previous steps were mixed thoroughly by pipetting. The microcarrier suspension was then supplied with 50 μl pre-chilled spermidine solution (1.45%, v/v) and mixed immediately by vortexing in the cold room (4°C) for 15–20 min. The plasmid-coated microcarriers were recovered by centrifugation (12,000 rpm for 10 s) and, following supernatant removal, were resuspended in 36 μl pure ethanol. For each bombardment, 10 μl gold suspension was loaded to the center of a macrocarrier disk (Bio-Rad), air-dried in the laminar flow hood, and placed in the microcarrier launch assembly under the 1100 psi rupture discs. Sixty immature embryos arranged in a 3.5-cm diameter circle were placed 6-cm below the macrocarrier assembly. The PDS-1000/He System was operated according to the manufacturer’s instruction. Bombardment conditions were 1,300 psi helium pressure and 25 mm Hg vacuum.

Putative transgenic plants were confirmed by PCR analysis. The presence of the selection marker (*BAR* or *HPT*) and other vector-specific fragments were tested in putative transgenic plants using PCR. Quantitative reverse transcription PCR (qRT-PCR) was used to quantify the downregulation of *FT1* transcription in the RNAi transgenic plants and to confirm the expression of the *FT1*_RNAi_ constructs. In addition, transgenic wheat plants were confirmed by testing their resistance to 0.3% (v/v) Finale herbicide.

### Mutant Screening and Genetic Analyses in Tetraploid Wheat

Genome-specific primers were designed for both *FT-A1* and *FT-B1* genes (Table 1) and were used to screen a tetraploid wheat TILLING population consisting of 1,384 EMS-treated individuals of the tetraploid wheat cultivar Kronos [31]. The screening was performed using a *Cel* I-based method described previously [31]. For the mutations described in this study, the first letter indicates the original base or amino acid and the last letter represents the mutant base or amino acid. The number in the middle indicates the position of the mutation counted from the ATG start codon in the genomic DNA sequence, or from the initial methionine in the predicted protein. For *FT-A1*, we selected mutant line T4-474 that carries a G655A mutation resulting in a premature stop codon. This mutant will be referred to hereafter as *ft-A1*. For *FT-B1* no truncation mutations were found, so two different mutations resulting in amino acid substitutions at conserved amino acids were selected from lines T4-263 (C856T) and T4-344 (G98A), designated hereafter as *ft-B1*_263_ and *ft-B1*_344_, respectively. The predicted amino acid changes in each of the mutant lines are described in the results section.

**Table 1:** PCR primers used in the current study.

Target	GenBank Acc.	Forward Primer (5′ to 3′)	Reverse Primer (5′ to 3′)	Efficiency	Objective	References
*TtFT-A1*	UCW_Tt_k51_contig_35084 1	TCGATCTACACTAGGAAGAAGGAAG	GTGGGCCATGGGTAGG	ND	TILLING	Currentstudy
*TtFT-B1*	UCW_Tt_k64_contig_13900 1	GTGGGGCAACACTCATCATC	GGCTGGTGGCGTACGAG	ND	TILLING	Currentstudy
*TtFT-A1*	UCW_Tt_k51_contig_35084 1	AGACGTGCTGGACCCCTTT	GACTTGGAGCATCTGGGTCT	98.50%	qRT-PCR	Currentstudy
*TtFT-B1*	UCW_Tt_k64_contig_13900 1	GGACGTGCTGGACCCCTTC	GACTTGGAGCATCTGGGTCT	99%	qRT-PCR	Currentstudy
*TaACTIN*	UCW_Tt-k41_contig_5677 1	ACCTTCAGTTGCCCAGCAAT	CAGAGTCGAGCACAATACCAGTTG	98%	qRT-PCR	[34]
*TaGI*	AY6791154	GAAGGTCAGAAGATGTGGAGAGTCAAC	GGCAGCGGATGGTAGGTGATAG	95%	qRT-PCR	[24]
*TaFT1*	CD881060	GCCGGTCGATCTATACTA	TCCTGTTCCCGAAGGTCA	101%	qRT-PCR	[24]
*TaFT2*	BT009051	TTTCTACACGCTGGTGATGG	GTGACCAGCCAGTGCAAGTA	96%	qRT-PCR	[21]
*TaFT3*	IWGSC_1AL_913428 2	GTACTTGCACTGGATGGTGTC	CATCTGGTGCAAAAACTGT	93%	qRT-PCR	Currentstudy
*TaFT4*	IWGSC_2AS_5252557 2	TGGATCCTGATGCGCCTAA	CAGTCACCATCCAGTGCAGGTA	109%	qRT-PCR	Currentstudy
*TaFT5*	IWGSC_5AL_2803506 2	ACGGTTTTTGCACCGGACA	GGCAGCGGCAATGTTGAG	100%	qRT-PCR	Currentstudy
*TaFT6*	IWGSC_6AS_4388307 2	GATATGCATGGCGGTTTCTC	CCAAGGAGTCGTTCGACATT	93%	qRT-PCR	Currentstudy
*TaVRN1*	JF965395	AAGAAGGAGAGGTCACTGCAGG	GGCTGCACTGCCGCA	99%	qRT-PCR	[7]
*TmACTIN*	AF326781	GCCATGTACGTCGCAATTCA	AGTCGAGAACGATACCAGTAGTACGA	99%	qRT-PCR	[33]
*BdGI*	Bradi2g05226	TACGGATGGGATGCTTGTTG	CGGCACTTCAGCAGATTCG	99%	qRT-PCR	Currentstudy
*BdFT1*	Bradi1g48830	CACACTACACACACGCAAGTACTGT	CAGCACGTCCCCCACAA	99%	qRT-PCR	Currentstudy
*BdFT2*	Bradi2g07070	TGGTTGTGATGGTCCGTTTG	AGACAGAACCGACTTGCTAGAAATTAC	98%	qRT-PCR	Currentstudy
*BdFT3*	Bradi2g49795	CCCTGGGACAACTGGAGCTA	TTCTTGGTTCTGGTCTTTCGTAGA	105%	qRT-PCR	Currentstudy
*BdFT4*	Bradi1g38150	TGGGCGGGAGATCGTAAC	CGGTGGATGCCCATGGT	106%	qRT-PCR	Currentstudy
*BdFT5*	Bradi2g19670	GAAGGTGGATCGGGTGGAA	GCTGTCTAGTCTTTACTCCCCTTGA	103%	qRT-PCR	Currentstudy
*BdFT6*	Bradi3g08890	GCGAGGACCTCAGCGTAACA	GCCGGGCTCTCGTAGCA	ND^3^	qRT-PCR	Currentstudy
*BdVRN1*	GT846767	GTCGCGCTCATCATCTTCTC	TGCATAGGAGTAGCGCTCATAG	100%	qRT-PCR	[23]
*BdACTIN*	Bradi4g41850	CCTGAAGTCCTTTTCCAGC	AGGGCAGTGATCTCCTTGC	99%	qRT-PCR	[53]

1 Sequences from tetraploid wheat Kronos [54];

2 Sequences from International Wheat Genome Sequencing Consortium (IWGSC) (Ensembl; http://plants.ensembl.org/index.html);

3 ND: Not Determined.

We backcrossed *ft-A1* three times to wild type Kronos and then crossed it with an M_3_ plant of the mutant line *ft-B1*_263_ to generate a BC_1_F_2_ population segregating for both mutations. Epistatic interactions between *FT-A1* and *FT-B1* were analyzed in this population using a factorial ANOVA. Data from this population was also used to calculate the degree of dominance for *FT-A1* and *FT-B1* using the formula: D = (2X_2_−X_1_−X_3_)/(X_1_−X_3_) [32] where X_1_, X_2_ and X_3_ are the heading time values, respectively, of the plants homozygous for the mutant late flowering allele (*ft*), the heterozygotes, and the plants homozygous for the wild type early flowering allele (*FT*). The degree of dominance for *FT-A1* was calculated using only *ft-B1*_263_ homozygous plants, and the degree of dominance for *FT-B1* was calculated using only *ft-A1* homozygous plants.

Mutant line *ft-B1*_344_ was backcrossed to Kronos and a separate BC_1_F_2_ segregating population was developed to evaluate the effect of this mutation on heading time. Both segregating populations were evaluated simultaneously for heading time in the same greenhouse experiment.

The transcript levels of *FT-A1* and *FT-B1* homoeologs were compared in three-week-old wild type Kronos plants grown in growth chambers under LD (16 h light, light intensity of 90 μmol m^−2^ s^−1^) at 16–20°C.

### Quantitative Reverse Transcription PCR (qRT-PCR) Analysis

Gene expression in *FT1*_RNAi_ transgenic plants was determined from six biological replicates. The youngest leaves of adult plants were collected at 12∶00 p.m. for *Brachypodium* and 4∶00 p.m. for hexaploid wheat. Total RNA was extracted from leaf tissues using the Trizol method (Life Technologies, Grand Island, NY, USA) according to the manufacturer’s instructions. cDNA templates were prepared using the Fermentas First Strand cDNA Synthesis Kit (Thermo Scientific, Waltham, MA, USA). In total, eight target genes were studied (Table 1). Ten-fold serial dilutions of cDNA templates were used to test the amplification efficiency of each primer pair. In *Brachypodium* and in Bobwhite transgenic wheat and control lines, qRT-PCR was performed using the FastStart SYBR Green Master (Roche Applied Science, Indianapolis, IN, USA) on the StepOnePlus Real-Time PCR Systems (Life Technologies). The amplification conditions were one cycle of 10 min at 95°C, 40 cycles of two consecutive steps of 15 s at 95°C and 1 min at 60°C and a standard dissociation protocol. *ACTIN* was used as an endogenous control using primers designed by Fu, et al. (Table 1; [33]).

In tetraploid wheat and in *FT1*_HOPE_ lines, the youngest leaf was collected at 10∶00 a.m. and RNA was extracted using the Spectrum Plant Total RNA Kit (Sigma-Aldrich, St. Louis, MO, USA). cDNA was synthesized using the High Capacity cDNA Reverse Transcription kit (Life Technologies) and used in qRT-PCR reactions performed on a 7500 Fast Real-Time PCR System (Life Technologies). Transcript levels of *FT-A1* and *FT-B1* homoeologs in tetraploid wheat were compared using genome specific primers (Table 1). *ACTIN* was used as an internal control using primers originally designed by Uauy, et al. (Table 1; [34]). The qRT-PCR conditions were one cycle of 20 s at 95°C and 40 cycles of two consecutive steps of 3 s at 95°C and 30 s at 60°C.

Transcript levels of target genes were calculated using the formula 1000*2^(*ACTIN* CT – *TARGET* CT)^, which indicates the relative number of target molecules per 1000 molecules of *ACTIN*. The expression data was analyzed using SAS version 9.0 (SAS Institute Inc, Cary, NC, USA). Data that did not meet the assumptions of the ANOVA was transformed using power transformations to restore homogeneity of variances (Levene’s test) and normality of residuals (Shapiro-Wilk test). Independent transgenic events were compared with the wild type control using Dunnett’s tests, and the information was summarized using a contrast comparing the wild type versus all transgenic lines. Graphs were prepared using the GraphPad Prism version 5.01 (GraphPad Software, San Diego, CA, USA).

### Pollen Staining

The viability of pollen grains in *FT1* transgenic plants was evaluated using a simplified staining protocol [35]. Mature but non-dehiscent anthers were collected and submerged in 100 μl staining solution for 24 h at room temperature and in darkness. After rinsing in distilled water, anthers were transferred to a fresh slide containing a drop of distilled water, and then inspected under the stereomicroscope. To measure the percentage of viable pollen, anthers were gently pressed to release pollen grains. Pollen grains stained magenta-red were viable, and those stained blue-green were non-viable. Two-hundred pollen grains within the microscopic view, performed in triplicate, were used to calculate the proportion of viable pollen.

## Results

### Overexpression of *FT1* Promoted Floral Organogenesis During Tissue Culture

**Figure 2:**
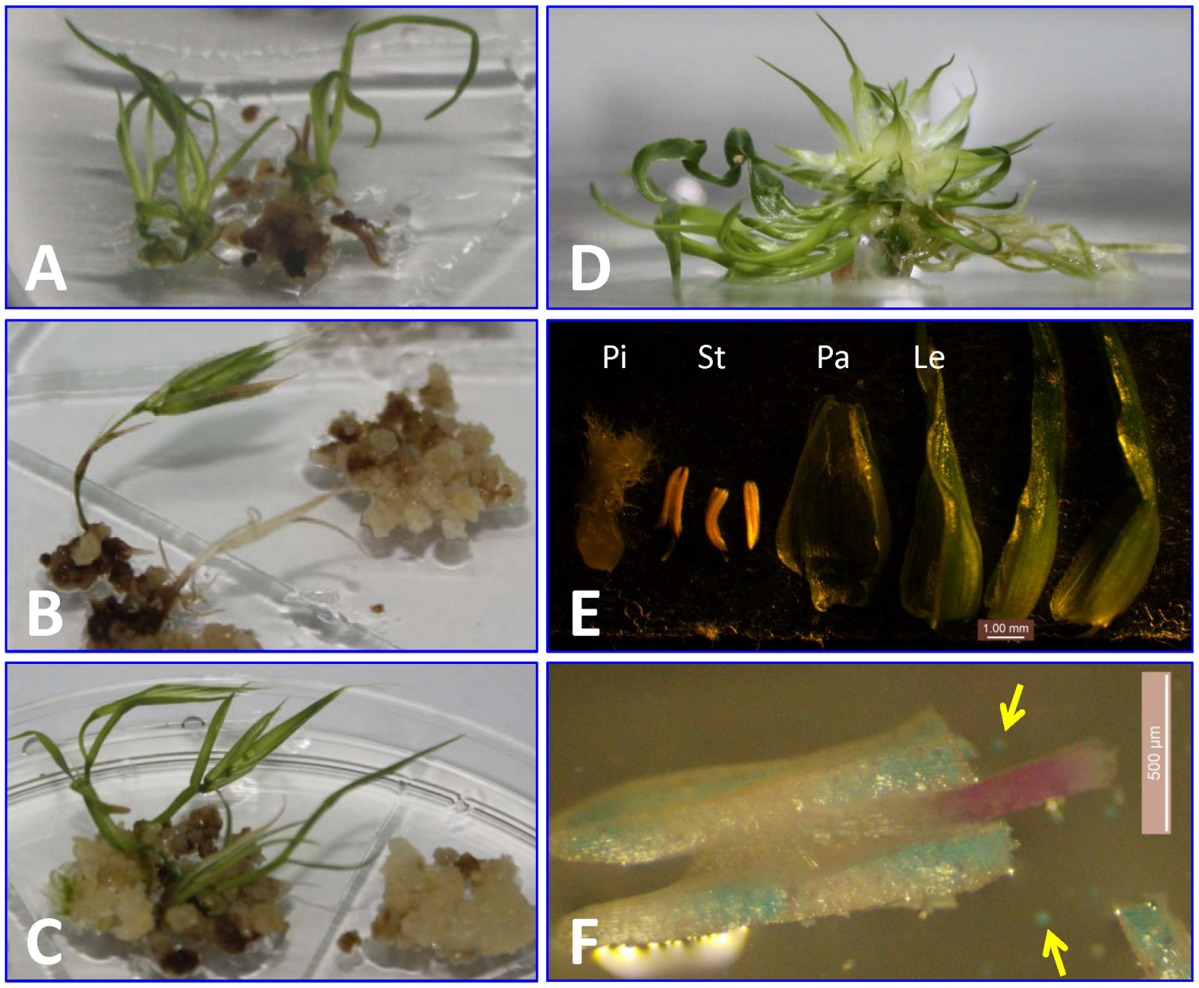
*FT1* overexpression promotes floral organogenesis. (A–C) *Brachypodium 35S::BdFT1:GFP*, (A) Shoot regeneration of non-transgenic control calli (B) direct spike formation from transformed calli and (C) rudimentary leaves associated with spikelet formation from transformed calli. (D–F) Wheat *Ubi::cFT-B1*. (D) Cluster of florets surrounded by rudimentary leaves in a transformed callus. (E) Different floral organs: lemma (Le), palea (Pa), pistil (Pi) and stamen (St). The additional organs seem to be glumes but it was difficult to determine because of the close clustering of multiple florets. (F) Anther with regions of non-viable pollen (blue color after pollen staining).

To study the effect of increased *FT1* expression on floral development, we developed transgenic *Brachypodium* and wheat plants overexpressing *FT1*. In the *Brachypodium* transformation experiment using the *FT1*_OE_ construct for Bd21-3 *FT1*cDNA driven by the maize *UBIQUITIN* promoter (Figure 1A), none of the 180 calli regenerated under LD (16 h light/8 h dark) and only two out of 100 calli regenerated under SD (8 h light/16 h dark), grew weakly, and eventually died. In the second *Brachypodium* transformation experiment using the fusion construct of Bd21-3 *FT1* and *GFP* driven by the 35S promoter (Figure 1B), six of 280 infected calli showed regenerated shoots that immediately developed floral organs under LD (Figure 2A–C). As a result of the limited development of vegetative tissue, none of the T_0_ plants produced seeds, and they did not survive when transplanted to soil. We then adjusted the light conditions during callus differentiation and shoot regeneration to a SD photoperiod and weak light intensity (29 μmol m^−2^ s^−1^). Under these conditions, the floral organogenesis was delayed and there was an increase in vegetative growth, but it was still insufficient to support the production of seeds. The lack of normal vegetative tissue also precluded the use of the GFP tag to study the localization of the FT protein in *Brachypodium* leaves.

In common wheat, we tested two different *FT1*_OE_ constructs, both driven by the maize *Ubiquitin* promoter: the first one using the *FT-B1* coding region (*Ubi::cFT-B1*) and the second one using the *FT-B1* genomic region (*Ubi::gFT-B1*) from tetraploid wheat Langdon (Figure 1A). Of the 1,876 calli bombarded with the *Ubi::cFT-B1* construct, 12 developed floral organs during culture. Of the 523 calli bombarded with the *Ubi::gFT-B1* construct, three flowered during the culture stage. PCR analysis confirmed that all early-flowering T_0_ plants were positive for the presence of the transgene (data not shown). In both cases, calli were maintained in recovery culture under dark conditions for four weeks and were then transferred to regeneration media under LD. Green shoots appeared five days later, and clusters of florets developed 3 to 4 weeks later (Figure 2D). At this stage, florets were opened wide and exhibited a normal feathery stigma but with smaller and shriveled stamens. Most florets had complete structures (including lemma, palea, pistil and stamens, Figure 2E), but some florets did not develop stamens. In comparison to wild type plants, the anthers of the transgenic plants had fewer pollen grains and a large proportion of non-viable pollen, as determined by pollen staining (Figure 2F). Similar to the transgenic *Brachypodium* plants, there was limited leaf development and none of the T_0_ plants produced seeds in culture or were able to survive when transplanted to soil.

### Downregulation of *FT1* Delayed Heading Time of Transgenic Plants

**Figure 3:**
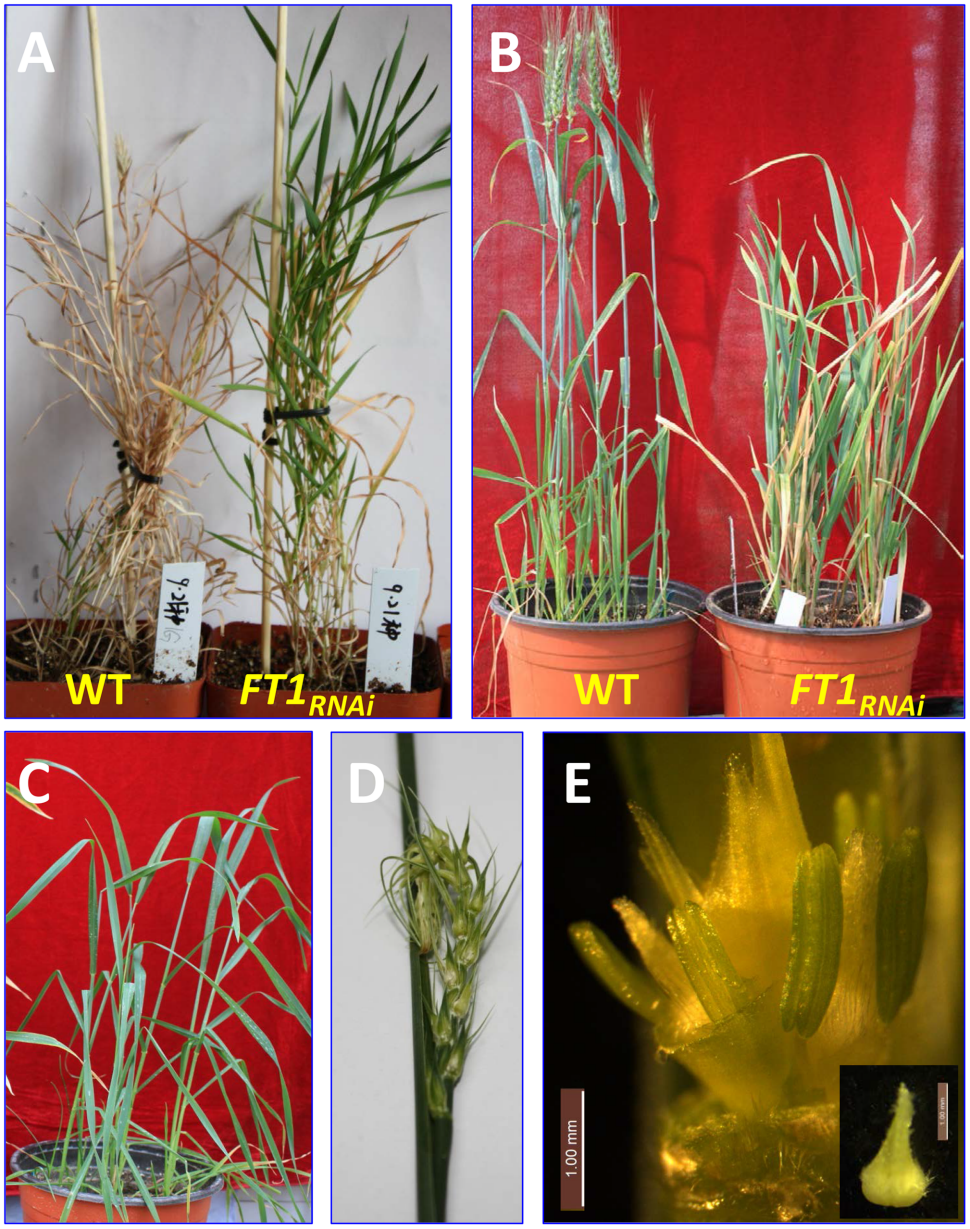
Silencing of *FT1* by RNAi delays heading time. (A) Heading was prevented in transgenic *FT1*_RNAi_ *Brachypodium* and (B) delayed in *FT1*_RNAi_ transgenic wheat. (C) Wheat transgenic plants at booting stage. (D) Some spikes had difficulty in emerging from the leaf sheath (leaf sheath opened manually in this picture). (E) Complete floral organs from transgenic wheat flowers (stigmas failed to open in some transgenic plants).

To determine the effect of reduced *FT1* expression on flowering development, we developed *Brachypodium* and wheat *FT1*_RNAi_ constructs that target only the *FT1* gene. Of the 280 *Brachypodium* calli transformed with the *Brachypodium FT1*_RNAi_ construct (Figure 1C), a total of 38 calli generated putative transgenic plants. PCR analyses demonstrated that all putative transgenic plants were positive for the *FT1*_RNAi_ construct. All *Brachypodium* transgenic T_0_ plants grew vigorously, but failed to flower (Figure 3A) even after two weeks of vernalization at 4°C, which is sufficient to induce flowering in the non-transgenic Bd21-3 control plants.

In the common wheat cultivar Bobwhite, the *FT1*_RNAi_ construct shares a continuous stretch of at least 179 identical nucleotides with the *FT-A1*, *FT-B1*, and *FT-D1* homoeologs, and therefore, is expected to silence all three copies simultaneously. Of the 1,675 wheat calli bombarded with the *FT1*_RNAi_ construct, 13 independent plants were confirmed to be transgenic from their resistance to the herbicide Finale and by PCR analysis. Transgenic lines showed heterogeneity in the effect of the transgene on heading time, floral organ morphology and fertility (Figure 3B–D, Table 2). Ten of the 13 transgenic plants showed moderate delays in heading time ranging from 2 to 4 weeks, two showed very late heading time (lines ‘1965’ and ‘2547’), and one (line ‘2548’) remained vegetative for ten months, before dying without flowering. Spikelets in line ‘2547’ had two complete florets, but additional florets were undeveloped. Of the two intact florets, each consisted of one pistil and three stamens, but the stigmas remained closed (Figure 3E) and the anthers stayed green and did not shed pollen. Of the two late transgenic lines only ‘1965’ set seeds.

**Table 2:** Heading date and floral characteristics of wheat *FT1*_RNAi_ transgenic plants.

Plant ID	Generation	Heading Date	Spikelets	Stigma	Anthers	Pollen Viability	Seed Setting
Bobwhite	WT	Normal	Normal	Bifid feathery	Normal	90–98%	Normal
Ten T_0_ lines^a^	T_0_	+14 to 28 d	Normal	–	–	–	Yes
1965	T_0_	+4 m	Normal	Variable^d^	Small	–	Yes
2547	T_0_	+6 m	Variable^c^	Closed	Small	–	None
2548	T_0_	Stay green	–	–	–	–	None
Three T_1_ lines^b^	T_1_	Normal	Normal	Normal	Normal	–	Yes
140D-1	T_1_	+14 d	Normal	Variable^d^	Small	47–66%	Yes
152E-1	T_1_	+14 d	Normal	Variable^d^	Small	43–78%	Yes
152E-2	T_1_	+17 d	Normal	Variable^d^	Small	11–32%	Yes
1965–2	T_1_	+10 d	Normal	Variable^d^	Small	45–67%	Yes
1965–5	T_1_	+20 d	Variable^c^	Variable^d^	Small	79–90%	None

a The ten T_0_ lines include ‘9′, ‘78A’, ‘116’, ‘140D’, ‘152E’, ‘157B’, ‘1381’, ‘1382’, ‘1384’ and ‘1828’;

b The three T_1_ lines include ‘157B’, ‘1381’ and ‘1382’;

c The first and second florets are normal, others deficient;

d Stigmas are bifid and feathery but open less widely than in the wild type.

Progeny tests including 5 to 10 T_1_ plants were performed for six independent transgenic lines, including the late-flowering line ‘1965’ and five moderately late-flowering lines. The T_1_ progeny of the late-flowering T_0_ transgenic plant ‘1965’ were not as late as the original T_0_ plant (only 10–20 days delay compared to the wild type) suggesting that late flowering of the T_0_ plant was not determined solely by the downregulation of *FT1*. Unfortunately, no seeds were available for the T_0_ transgenic lines showing the greatest delay in flowering (lines ‘2547’ and ‘2548’) and, therefore, we were unable to verify the linkage between these late flowering phenotypes and the transgene.

Progeny for lines ‘140D’, ‘152E’ and ‘1965’ segregated for early and late heading time (14 to 20 days later than the Bobwhite control). Progeny of ‘152E’ developed relatively normal florets and set seeds. In a few lines, e.g. ‘140D-1′ spikes failed to emerge from the sheath of the flag leaf resulting in abnormal curling of the spike and awns (Figure 3D). Additionally, the florets developed pistil and stamen, but some florets had abnormal stigmas which failed to open after heading and did not set seeds. All plants from the progeny of lines ‘157B’, ‘1381’ and ‘1382’ headed at the same time as the wild type control plants suggesting that they were not functional transgenic lines (Table 2). In summary, *FT1*_RNAi_ transgenic wheat lines flowered 2 to 4 weeks later than the wild type control, exhibited reduced pollen viability and in some cases mature stigmas did not open as widely as those in control plants (Table 2).

### Mutations in *FT-A1* and *FT-B1* Delay Flowering in Tetraploid Wheat

We screened a TILLING population of the tetraploid wheat cultivar Kronos and identified 38 mutant alleles for *FT-A1* and 13 for *FT-B1*, and selected three for functional characterization (Table 3). The G655A mutation present in the selected *ft-A1* mutant disrupts the splice site located at the beginning of the second intron. Sequencing of the resulting *ft-A1* cDNA using homoeolog-specific primers confirmed the elimination of the splice site, which resulted in a 4-bp insertion and an in-frame premature stop codon at position 88 (W88*). This premature stop codon eliminates the last 90 amino acids (50.8% of the protein), and almost certainly produces a non-functional protein.

**Table 3:** Summary of the *ft1* mutants in tetraploid wheat cultivar Kronos.

Line	Gene	Nt Mutation^a^	Pr Mutation b	BLOSUM 62^c^	*At/Os/Zm* d	*Hv/Ta/Tm/Tt* d				*Hv/Tt* d	
					FT1	FT1	FT2	FT3	FT4	FT5	FT6
T4-474	*TtFT-A1*	G655A	W88*	N/A	W	W	W	W	W	W	W
T4-263	*TtFT-B1*	C856T	P77S	−1	P	P	P	P	P	P	P
T4-344	*TtFT-B1*	G98A	G33E	−2	G	G	A	N	D	N	D

a Mutations in the DNA (Nt, position is relative to the start codon ATG);

b Mutations in the predicted protein (Pr) (counted from the initial methionine);

c BLOSUM 62 scores [36];

d Amino acid present in other species: *Arabidopsis* (*At*), *Hordeum vulgare* (*Hv*), *Oryza sativa* (*Os*), *Triticum aestivum* (*Ta*), *T. monococcum* (*Tm*), *T. turgidum* (*Tt*), and *Zea mays* (*Zm*). All homoeologous alleles were included in the comparison for the polyploidy wheat species.

No truncations or splice site mutations were identified for *FT-B1*, so two substitution mutations within the PEBP motif were selected based on high evolutionary conservation of the targeted amino acid and negative BLOSUM62 scores, which are predictive of altered structural and functional properties [36]. In mutant line *ft-B1*_263_, a C856T mutation resulted in a change between proline and serine (P77S, BLOSUM62 score = −1). The proline at position 77 is highly conserved in FT1 orthologs in Arabidopsis, maize, rice, diploid, tetraploid and hexaploid wheat and in all six FT-like proteins (FT1 to FT6) in barley and wheat (Table 3). Mutant line *ft-B1*_344_ carries a G to A mutation at position 98 (G98A), which results in the amino acid change G33E (BLOSUM62 score = −2). The original amino acid (glycine) is conserved in the FT1 orthologs in Arabidopsis, maize, rice, barley, diploid, tetraploid and hexaploid wheat. In the other FT-like proteins this amino acid position is substituted by alanine, asparagine or aspartic acid, but in no case by glutamic acid as in the G33E mutation (Table 3).

To quantify the effect of the *ft-A1* truncation mutation, the *ft-B1*_263_ substitution mutation, and their interaction we genotyped 110 plants from a BC_1_F_2_ population segregating for these two mutations and determined their heading time under LD without vernalization (Kronos has a spring growth habit). The wild type alleles for early flowering showed partial dominance over the mutant alleles, with an estimated degree of dominance of approximately 0.46 and 0.59 for *FT-A1* and *FT-B1*, respectively.

**Figure 4:**
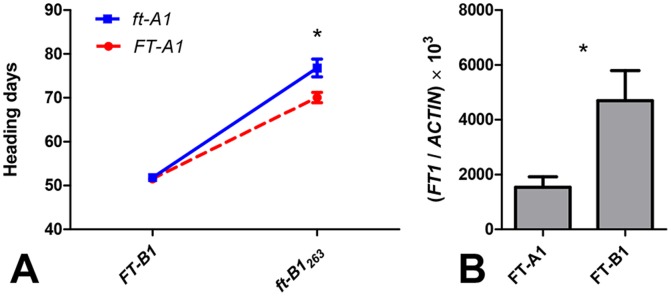
*ft1* TILLING mutants. (A) Interaction plot showing the effect of *ft-A1* and *ft-B1*_263_ mutations on heading time in a BC_1_F_2_ population segregating for both genes. Differences in heading time between the *FT-A1* alleles were significant only for the homozygous *ft-B1*_263_ mutant plants. (B) Transcript levels of *FT-A1* and *FT-B1* homoeologs in three-week-old wild type Kronos. The expression level of *FT-B1* was significantly higher than that of the *FT-A1* (*P* = 0.017). *ACTIN* was used as an internal control. Samples were harvested at 10∶00 a.m. Asterisks indicate *P* values of Student’s *t*-tests: * = *P*<0.05.

Based on this partial dominance we decided to merge the homozygous wild type and heterozygous classes into a single non-mutant class. This strategy has the advantage of simplifying the analysis and description of the interactions between these two genes, and of increasing the power of the statistical analyses. The resulting 2×2 factorial ANOVA (using *FT-A1* and *FT-B1* as factors) revealed significant differences in heading time both for *FT-A1* (*P = *0.0075) and *FT-B1* (*P<*0.0001), as well as a significant interaction (*P = *0.0143, Figure 4A) between the two genes. This interaction indicates that the effect of each *FT1* homoeolog on flowering time is dependent on the allele present in the other homoeolog.

To describe these interactions, we analyzed the simple effects of each *FT1* homoeolog within the two classes of the other *FT1* homoeolog. The *FT-B1* alleles showed highly significant differences in heading time (*P<*0.0001) within both alleles of *FT-A1*, but the differences between the adjusted means of the *FT-B1* alleles were 34% larger in the lines homozygous for the mutant *ft-A1* allele (25.0 day delay in flowering) than in the lines homozygous or heterozygous for the wild type *FT-A1* allele (18.6 day delay). The effects of the *FT-A1* alleles on heading time were smaller than those of the *FT-B1* alleles, and the differences were significant only within the homozygous *ft-B1*_263_ mutant class (6.7 day delay; *P* = 0.0374). In all cases, plants carrying mutant *ft1* alleles flowered later than plants homozygous or heterozygous for the wild type alleles. Plants homozygous for both *ft-A1* and *ft-B1*_263_ mutant alleles flowered on average 25.0 days later than plants homozygous or heterozygous for the two wild type alleles, confirming an important role of *FT1* in the acceleration of wheat flowering under LD.

To characterize the effect of the *ft-B1*_344_ mutation (G98A) on heading time we genotyped and phenotyped 23 BC_1_F_2_ plants segregating for the *ft-B1*_344_ mutation in a genetic background fixed for the wild type *FT-A1* allele. Plants homozygous for the wild type *FT-B1* allele headed on average in 43.5 days while plants homozygous for the mutant *ft-B1*_344_ allele headed in 51.2 days (7.7 day delay). This difference was smaller than that detected between homozygous lines of wild type and *ft-B1*_263_ mutation (20.4 day delay within the same homozygous wild type *FT-A1* background), suggesting that the *ft-B1*_344_ mutation has a less deleterious effect on *FT-B1* function than the *ft-B1*_263_ mutation.

The predicted proteins encoded by the *FT-A1* and *FT-B1* genes in tetraploid wheat are identical, so we hypothesized that the larger effect of the *ft-B1*_263_ mutant relative to the *ft-A1* truncation mutant on heading time could be associated with relative differences in their respective expression levels. To test this hypothesis, we designed genome-specific primers for *FT-A1* and *FT-B1* and quantified their transcript levels using qRT-PCR (Table 1) in three-week-old wild type Kronos plants. Transcript levels of the *FT-B1* homoeolog were three-fold higher than those of the *FT-A1* homoeolog (Figure 4B), which correlates with the stronger effect of the *FT-B1* locus on heading time.

### Expression of *FT*-like Genes in Transgenic *FT1* Plants

**Figure 5:**
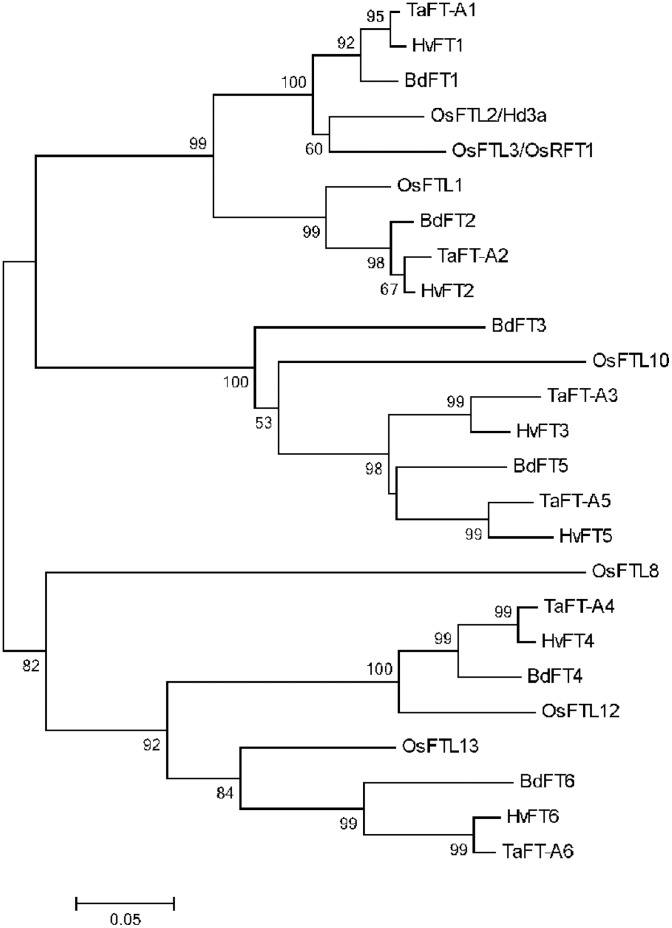
Phylogenetic analysis of *FT*-like genes in temperate grasses. Phylogenetic analysis was performed using the full-length proteins. A neighbor-joining tree was constructed using pairwise deletions and 1,000 bootstrap iterations with the program MEGA 5.0 [52]. The scale bar 0.05 represents 5% base substitution. Bootstrap numbers larger than 50 are shown in the respective nodes. To simplify the tree, only the wheat A-genome homoeologs of wheat were included. Accession information: *BdFT1* (*Bradi1g48830*), *BdFT2* (*Bradi2g07070*), *BdFT3* (*Bradi2g49795*), *BdFT4* (*Bradi1g38150*), *BdFT5* (*Bradi2g19670*), *BdFT6* (*Bradi3g08890*), *HvFT1* (DQ100327), *HvFT2* (DQ297407), *HvFT3* (DQ411319), *HvFT4* (DQ411320), *HvFT5* (EF012202), *HvFT6* (morex_contig_54196), *OsFTL1* (*Os01g11940*), *OsFTL2/Hd3a* (*Os06g06320*), *OsFTL3/RFT1* (*Os06g06300*), *OsFTL8* (*Os01g10590*), *OsFTL10* (*Os05g44180*), *OsFTL12* (*Os06g35940*), *OsFTL13* (*Os02g13830*), *TaFT-A1* (CD881060), *TaFT-A2* (BT009051), *TaFT-A3* (IWGSC_1AL_913428), *TaFT-A4* (IWGSC_2AS_5252557), *TaFT-A5* (IWGSC_5AL_2803506), *TaFT-A6* (IWGSC_6AS_4388307). *HvFT6* sequence is from the International Barley Sequencing Consortium (IBSC, http://webblast.ipk-gatersleben.de/barley/).

Based on the observation that the transcript levels of *FT2* were altered in transgenic wheat plants with higher transcript levels of *FT1* [21] we decided to investigate the effect of *FT1* down- and up-regulation on the five closest paralogs to *FT1.* To provide an evolutionary framework for the different wheat and *Brachypodium FT-*like genes included in the expression analyses, we first characterized their phylogenetic relationships (Figure 5). There are multiple *FT*-like genes in both *Brachypodium* and wheat [12], but only the closest five are included in this study. The predicted proteins from *Brachypodium*, wheat, barley and rice were grouped into five clusters. Both the FT1 and FT2 clusters include homologs from all four species, which suggests an ancient duplication that predates the divergence between rice and the temperate grasses. The FT3 and FT5 clusters are more closely related to each other than to the FT4 cluster, which includes two closely related proteins, FT4 and a previously undescribed protein, which we have named FT6 (Table 1). Barley and *Brachypodium* homologs of this novel FT protein were also identified (Figure 5).

**Figure 6:**
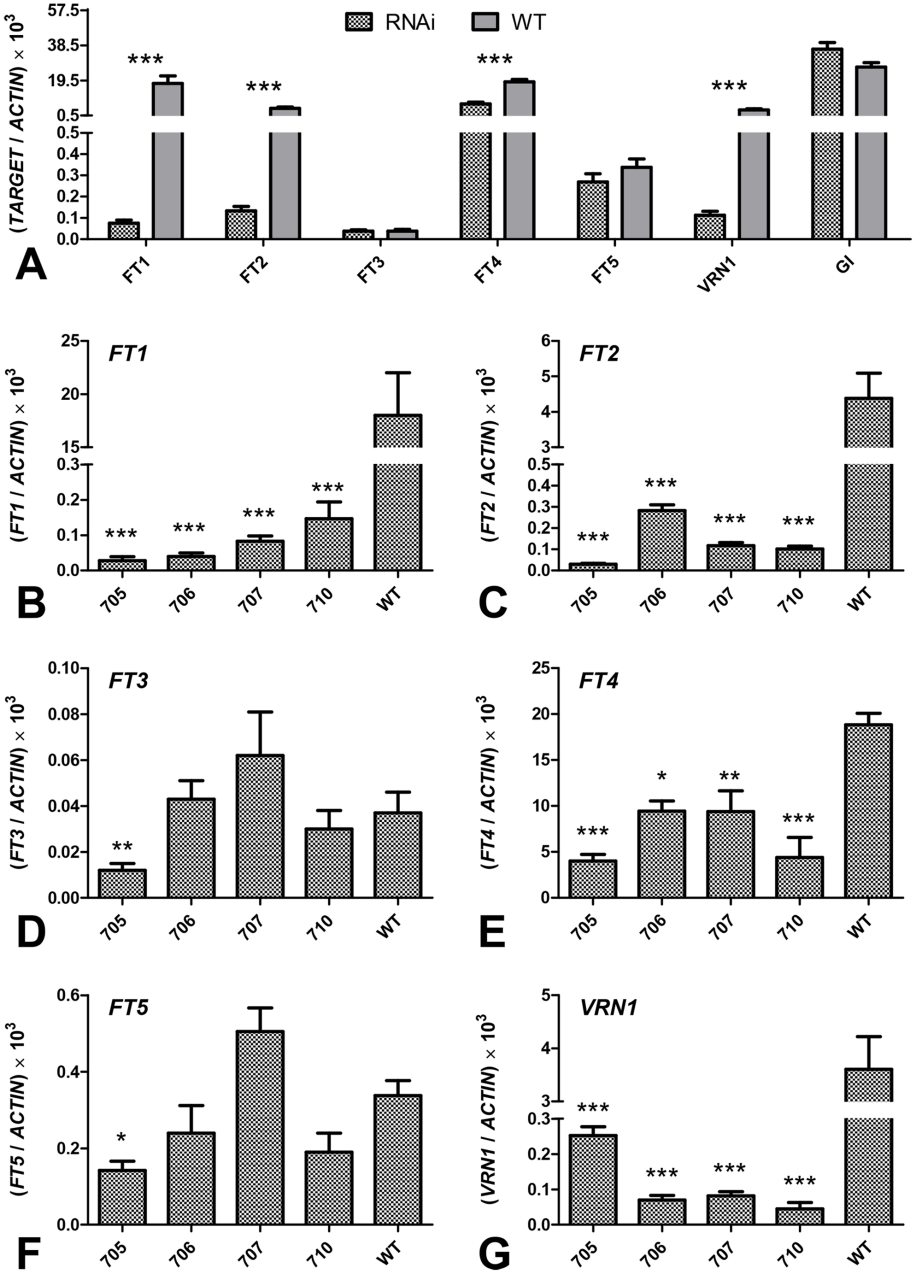
Transcript levels of target genes in T_0_ *Brachypodium FT1*_RNAi_ lines. (A) Comparison between the average of four transgenic plants (RNAi) and the wild type control using contrasts. (B–G) Comparison between individual transgenic lines and wild type using Dunnett’s test. (B) *FT1*, (C) *FT2*, (D) *FT3*, (E) *FT4*, (F) *FT5*, (G) *VRN1* (gene regulated by *FT1*). *ACTIN* was used as the internal control. Samples were harvested at 12∶00 p.m., approximately one week after wild type control plants began to flower. Asterisks indicate *P* values: * = *P*<0.05; ** = *P*<0.01; *** = *P*<0.001.

*Brachypodium FT1*_RNAi_ T_0_ plants ‘705’, ‘706’, ‘707’, and ‘710’ grown under LD showed a significant reduction in *FT1* transcript levels (<1%, *P<*0.001) relative to the wild type control (Figure 6A–B). The same transgenic plants also showed a significant downregulation of the *FT-*like genes *FT2* (average 3% of wild type, *P*<0.001, Figure 6C) and *FT4* (average 36% of wild type, *P*<0.001, Figure 6E), but their expression of *FT3* or *FT5* was not significantly different from the wild type (Figures 6A,D,F). The expression of *FT6* was nearly undetectable in both wild type and transgenic plants (data not shown). The *FT1*_RNAi_ transgenic plants also showed a strong downregulation of the *FT1* downstream target *VRN1* (∼3% of wild type, *P*<0.001, Figure 6G), which may explain the non-flowering phenotype of these lines. As expected, no significant effects were detected in the expression of *GIGANTEA* (*GI*) (Figure 6A), an upstream regulator of *FT* in the photoperiodic pathway in Arabidopsis [37].

**Figure 7:**
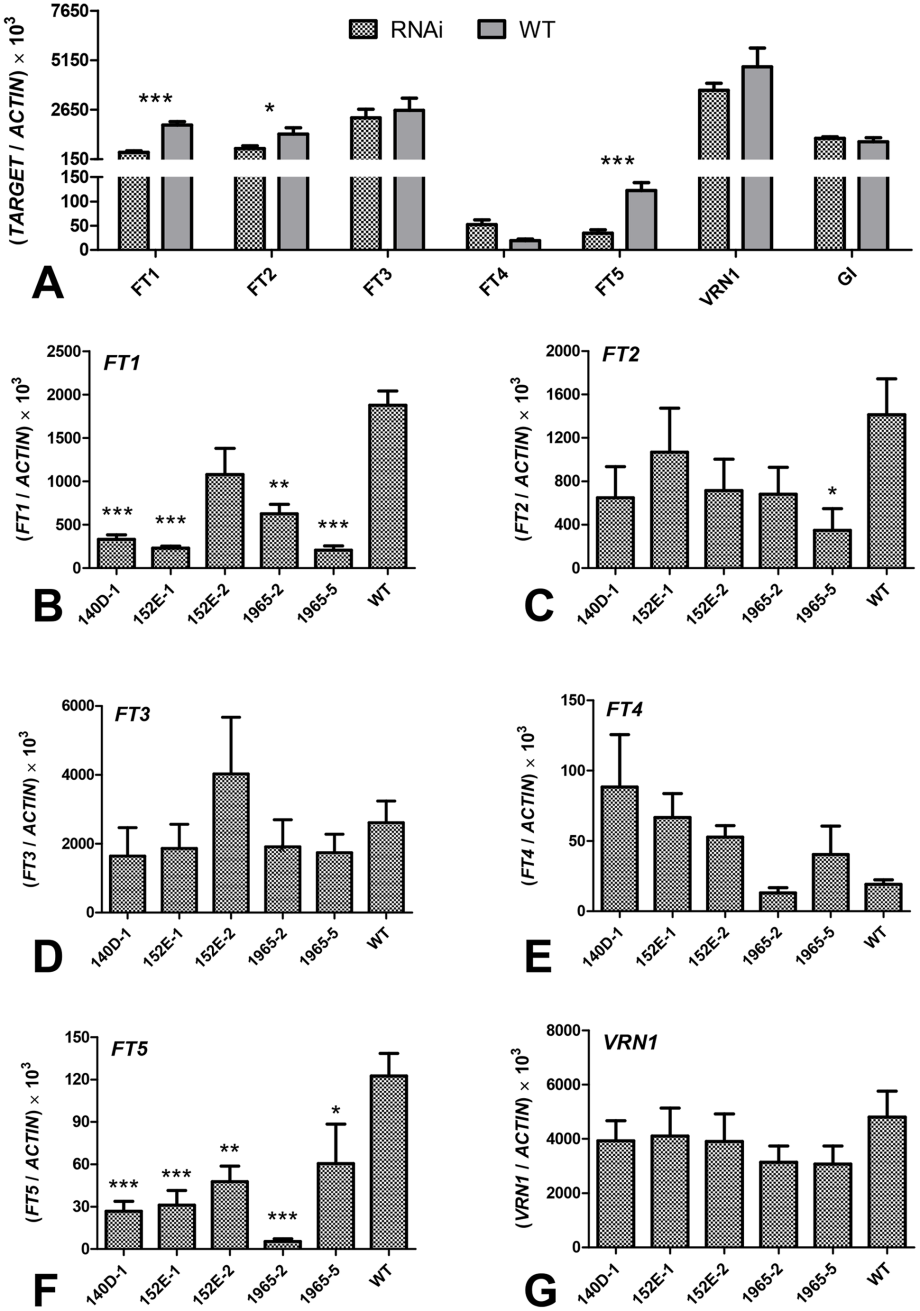
Transcript levels of target genes in transgenic T_1_ wheat RNAi plants and the non-transgenic wheat control. (A) Comparison between the average of five transgenic plants (RNAi) and the wild type control using contrasts. (B–G) Comparison between individual transgenic lines and wild type using Dunnett’s test. (B) *FT1*, (C) *FT2*, (D) *FT3*, (E) *FT4*, (F) *FT5*, (G) *VRN1* (gene regulated by *FT1*). *ACTIN* was used as the internal control. Samples were harvested at 4∶00 p.m., when the wild type controls began to flower. Asterisks indicate *P* values: * = *P*<0.05, ** = *P*<0.01, *** = *P*<0.001.

In the five selected wheat *FT1*_RNAi_ T_1_ transgenic lines (‘140D-1′, ‘152E-1′, ‘152E-2′, ‘1965-2′, and ‘1965-5′), *FT1* transcript levels were significantly reduced relative to the levels observed in the wild type Bobwhite (∼26%, *P*<0.001, Figure 7A–B). The downregulation of *FT1* was associated with significant reductions in the transcript levels of *FT2* (49%, *P*<0.05, Figures 7A,C) and *FT5* (28%, *P*<0.001, Figures 7A,F). No significant differences between mutant and wild type lines were detected for *FT-*like genes *FT3* and *FT4* (Figures 7A,D–E) or for the *GI* upstream control (Figure 7A). As in *Brachypodium*, the expression of the wheat *FT6* gene was also nearly undetectable in both wild type and transgenic plants (data not shown). Transcript levels of *VRN1* were slightly reduced (76% of wild type) in the transgenic plants, but these differences were not significant (Figures 7A,G). The smaller differences in *VRN1* transcript levels and in flowering time between the wheat *FT1*_RNAi_ and wild type compared with those observed in *Brachypodium*, might be associated with the presence of dominant *VRN1* alleles for spring growth habit in both the Bobwhite transgenic and control plants (*Vrn-A1, Vrn-B1* and *Vrn-D1*; [38]).

**Figure 8:**
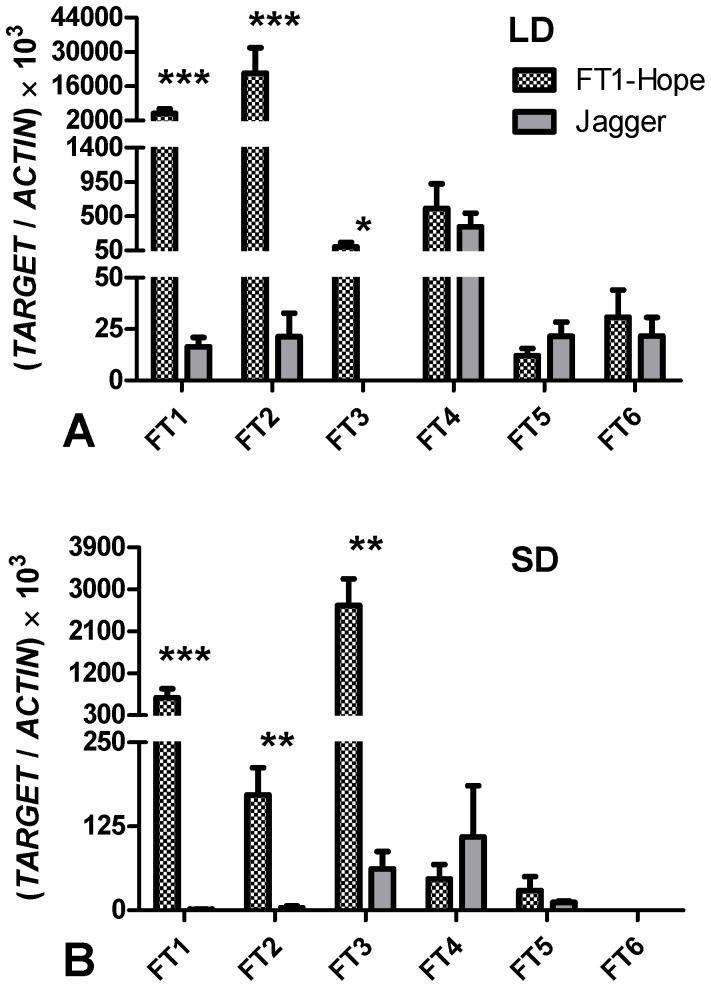
Expression of *FT*-like genes in *FT1*_HOPE_ transgenic wheat. The experiment was performed on transgenic lines (*FT1*_HOPE_) and the non-transgenic control Jagger. Leaf tissues were collected from two independent experiments, including plants grown under a (A) LD photoperiod for five weeks and (B) plants grown under a SD photoperiod for six weeks. *ACTIN* was used as the internal control. Samples were harvested at 10∶00 a.m. Asterisks indicate *P* values: * = *P*<0.05, ** = *P*<0.01, *** = *P*<0.001.

To further test the effect of *FT1* (or of the developmental changes induced by *FT1*) on the expression of other *FT-*like genes, we quantified the transcript levels of these genes in wheat transgenic plants carrying the highly-expressed Hope *FT-B1* allele [7]. In the transgenic *FT1*_HOPE_ lines, the significant increase in *FT1* transcript levels was associated with a significant upregulation of *FT2* and *FT3* both under LD (Figure 8A) and SD (Figure 8B) photoperiods. Expression levels of *FT4*, *FT5* and *FT6* were not significantly different between the wild type and *FT1*_HOPE_ lines. Taken together these results indicate that the developmental changes induced by *FT1* are associated with changes in the expression of other *FT-*like genes.

## Discussion

### *FT1* is a Strong Promoter of Flowering Initiation in Both *Brachypodium* and Wheat

In the temperate grasses, the shoot apical meristem is located at the base of the plant covered by multiple layers of nested leaf primordia, sometimes below the soil and/or a layer of dead leaves, and is thus relatively isolated from some environmental signals. In contrast, leaves are broadly exposed and act as the main sensory organ for monitoring changes in the environment. *FT1* and possibly other *FT*-like genes play a central role in the integration and transport of these signals [7], [18]. The convergence of multiple signaling pathways in the regulation of these genes is likely related to the distinctive ability of their encoded proteins to move through the phloem transporting environmental signals from the leaves to the shoot apical meristems [17], [20].

In this study we show that the ectopic expression of *FT1* under constitutive promoters results in precocious flowering of differentiating wheat and *Brachypodium* calli, bypassing the generation of normal vegetative tissues. Similar results have been reported before for both *Brachypodium* and wheat. Two recent studies have shown that overexpression of *FT1* (and also of *FT2*) in *Brachypodium* results in precocious flowering [25], [26]. Similarly, transformation of the *FT-D1* wheat homoeolog driven by the 35S promoter into the spring wheat variety ‘Norin 61′ [24], or of a highly expressed *FT-B1* allele driven by its native promoter into the winter wheat Jagger (*FT1*_HOPE_) [7], accelerate wheat heading time. Taken together, these results demonstrate that *FT1* is a strong activator of the flowering pathway in both wheat and *Brachypodium*.

*FT1* has the ability to induce the transcription of *VRN1* meristem identity gene even in the absence of vernalization. In varieties with a winter growth habit, chromatin in *VRN1* regulatory regions is in a repressed state before vernalization and cold treatment promotes a more active state leading to the upregulation of *VRN1* [39]. We show here that the overexpression of *FT1* in the vernalization-responsive *Brachypodium* accession Bd21-3 [26], [40] results in extremely early flowering, even in the absence of vernalization. Similarly, the introgression of natural *FT1* alleles expressed at high levels in early developmental stages for both wheat [7] and barley [41] into varieties with a winter growth habit also results in early flowering in the absence of vernalization.

### Downregulation of *FT1* Showed Stronger Effects in *Brachypodium* than in Wheat

Results from the *FT1* overexpression experiments presented here and from previous studies [7], [24], [25] indicate that the expression of *FT1* is sufficient to induce flowering in both *Brachypodium* and wheat. However, these studies have not described the effect of the downregulation of *FT1*, which is important to determine if this gene is essential for flowering in the temperate cereals. In *Brachypodium FT1*_RNAi_ transgenic lines, the drastic downregulation of *FT1* was associated with a non-flowering phenotype, even after the plants were vernalized for two weeks. This result indicates that *FT1* is essential for flowering in this *Brachypodium* line under the conditions tested in this study.

Rice *FT1* homologs also seem to be essential for flowering [42]. In this species, there are two duplicated *FT1* orthologs, *Hd3a* and the *Rice Flowering Locus T1* (*RFT1*) that share 90% identity at the protein level, suggestive of a relatively recent duplication in the rice lineage [42]. The simultaneous silencing of *Hd3a* and *RFT1* by RNAi results in non-flowering rice plants, even when grown in an inductive SD photoperiod [42]. In contrast, non-functional mutations in all six *FT-*like genes in Arabidopsis delay, but do not abolish flowering [43]. These studies in rice and Arabidopsis indicate that the importance of *FT-*like genes in flowering regulation carries across species. Our *Brachypodium* and wheat results support this conclusion. While *FT1* seems to be essential for flowering in *Brachypodium,* in wheat the downregulation by RNAi or disruptive mutation in the coding regions result only in delayed flowering.

In wheat, most *FT1*_RNAi_ transgenic plants (10 out of 13 T_0_ lines) flowered only two to four weeks later than the wild type Bobwhite plants (Table 2) and were able to set seeds. Among the three extremely late or non-flowering wheat *FT1*_RNAi_ transgenic lines only one set seed (line ‘1965’). A progeny test of this line showed that the derived T_1_ progeny flowered 10 to 20 days later than the wild type, similar to the other transgenic lines (Table 2). This result suggests that other factors (e.g. tissue culture) may have contributed to the later flowering time of the ‘1965’ T_0_ plant relative to its T_1_ progeny. The other two late flowering wheat plants (lines ‘2547’ and ‘2548’) failed to produce seeds and, therefore, it was not possible to verify whether their unusually late flowering phenotype was linked to the *FT1*_RNAi_ transgene.

The stronger effect of the *FT1*_RNAi_ transgene on flowering time in *Brachypodium* relative to wheat was correlated with a stronger downregulation of *FT1* in the *Brachypodium* transgenic lines (<1% of wild type) than in wheat (∼26% of wild type). We currently do not know if this is caused by differences in the efficiency of the RNAi constructs, or by the different genetic backgrounds. The Bd21-3 line selected for *Brachypodium* transformation has a facultative winter growth habit [40] whereas the Bobwhite genotype used in wheat transformation has a spring growth habit. The *Vrn-A1, Vrn-B1* and *Vrn-D1* alleles present in Bobwhite are dominant for early flowering and epistatic to the winter alleles of other vernalization genes. These *VRN1* alleles may contribute, directly or indirectly, to the relatively higher *FT1* transcript levels observed in the wheat RNAi lines than in the *Brachypodium* RNAi lines.

The tetraploid line Kronos used to generate the *ft1* TILLING mutants also carries a *Vrn-A1* allele that is dominant for spring growth habit and epistatic to other vernalization genes [44]. This may contribute to the limited effect of the *ft-A1/ft-B1*_263_ double mutant, which showed a flowering delay (25 days) similar to that observed in the *FT1*_RNAi_ transgenic hexaploid wheat plants. Unfortunately, we were unable to find a truncation mutation for the *FT-B1* homoeolog and therefore, we cannot rule out the possibility that the selected *ft-B1*_263_ mutant has some residual ability to induce flowering.

Triple *ppd1*-null mutants in the photoperiod sensitive hexaploid spring variety ‘Paragon’, which have almost undetectable *FT1* transcript levels, flower approximately 30 days later than their wild type control [45]. This delay in flowering is similar to that observed in the *ft-A1/ft-B1*_263_ double mutant and in the Bobwhite *FT1*_RNAi_ transgenic lines. Taken together, these results support the hypothesis that *FT1* is not essential for flowering in spring wheat. Even if other genes can induce flowering in wheat, our results demonstrate that *FT1* still plays a critical role in the timely induction of reproductive development in both *Brachypodium* and wheat.

### Changes Induced by *FT1* are Associated with Changes in Other *FT-*like Genes

*FT-*like genes *FT2*, *FT3*, *FT4*, *FT5* and *FT6* are the closest paralogs of *FT1* in the temperate grasses [12]. Among these, *FT2* is the most similar to *FT1* (>75% protein identity), and is part of a duplication that occurred before the divergence of the rice and wheat lineages (Figure 5). The FT3 and FT5 clusters (80 to 84% protein identity) appear to have arisen from a more recent duplication. Wheat and barley FT3 and FT5 proteins form well supported clusters, but their relationship with the *Brachypodium* homologs is unclear. This suggests that this duplication occurred prior to the divergence of wheat and barley, but close to the time of divergence between the *Brachypodium* and the *Triticeae* lineages. A separate cluster includes two groups of wheat, *Brachypodium* and barley paralogs; the FT4 group that is related to rice OsFTL12 and the FT6 group that is related to rice OsFTL13 (Figure 5). The FT4 and FT6 paralogs have a similar level of divergence (71 to 75% protein identity) to that between the FT1 and FT2 paralogs.

Downregulation of *FT1* in the *FT1*_RNAi_ transgenic plants in *Brachypodium* and wheat results in a significant downregulation of *FT2*, while upregulation of *FT1* in *FT1*_HOPE_ wheat plants results in a significant upregulation of *FT2*. These results suggest that, directly or indirectly, *FT2* transcription is regulated by *FT1*. Overexpression of *FT2* homologs in *Brachypodium* [25] and rice (*OsFTL1*) [46] results in extremely early flowering similar to the results observed for *FT1*. These results suggest that *FT1* and *FT2* paralogs have both retained the ability to induce flowering. Therefore, the changes in *FT2* transcript levels observed in the *FT1*_RNAi_ and *FT1*_HOPE_ transgenic plants may also contribute to the observed differences in flowering time.

Significant changes in the expression of other *FT-*like genes were detected in the *FT1*_RNAi_ and *FT1*_HOPE_ transgenic plants, but these effects were less consistent across genotypes and environmental conditions. The association between reduced *FT1* transcript levels in the *FT1*_RNAi_ lines and the downregulation of *FT4* was significant only in *Brachypodium,* whereas the association with the downregulation of *FT5* was significant only in wheat. While the effect of *FT1* on *FT3* expression was significant in both LD and SD grown *FT1*_HOPE_ transgenic plants, the transcript levels of wheat *FT3* were 25-fold higher under SD than under LD (Figure 8). This has also been reported previously in barley, where *FT3* is a candidate gene for a major QTL affecting barley flowering under SD (*Ppd-H2*) [12]. Overexpression of barley *FT3* in rice also accelerates flowering [47]. However, this effect is significantly smaller than the acceleration of flowering time in rice transgenic lines transformed with the barley *FT1* and *FT2* genes and is modulated by photoperiod [47]. These results suggest that *FT3* may play a role distinct from that of *FT1* and *FT2* in the regulation of flowering time.

No significant differences in *FT4, FT5* or *FT6* transcript levels were detected between Jagger and the Jagger *FT1*_HOPE_ transgenic line, but the transcript levels of these three genes were higher under LD than under SD in the wild type Jagger plants suggesting some role in the photoperiodic response (Figure 8). The roles of *FT4*, *FT5* and *FT6* and their rice homologs remain poorly understood.

The observed reduction in the expression of *FT-*like genes in the *FT1*_RNAi_ transgenic plants is unlikely to be the result of a direct targeting of the RNAi construct, because the region of *FT1* selected as target for RNAi did not share any stretches of more than 18 identical nucleotides with other *FT*-like genes. A continuous stretch of at least 21 identical nucleotides between the trigger and a target gene is required to produce efficient silencing [48]. Therefore, the transcriptional changes observed in other *FT-*like genes in the leaves of the transgenic plants are likely a result of the changes in *FT1* or of the developmental changes induced by *FT1*. Additional studies will be required to validate the effect of *FT1* on the expression of other *FT*-like genes.

In rice, co-silencing of the *FT1* homologs *Hd3a* and *RFT1* also resulted in the downregulation of the *FT2* homolog *OsFTL1*, but showed no effect on the expression of the rice homologs of *FT3* and *FT5* (*OsFTL10*) or *FT4* (*OsFTL12*) [42]. Therefore, the observed changes in *FT4* and *FT5* transcription could be species specific, or be affected by different developmental stages of the plants in the different experiments.

The fact that FT1 interacts with the bZIP transcription factors FDL2 and FDL6 (which can bind the *VRN1* promoter), while FT2 interacts with FDL13 (which cannot bind the *VRN1* promoter) suggests that FT1 and FT2 may have different gene targets [21]. This observation is consistent with the functional divergence observed for the FT1 and FT2 orthologs in rice (Hd3a and OsFTL1). Whereas overexpression of *Hd3a* in rice results in an early flowering phenotype [49], overexpression of *OsFTL1* produces a more complex phenotype including elongation of internodes, loss of apical dominance, and formation of a terminal tissue at the apical meristem [46].

In rice, Hd3a forms a hexameric protein complex comprised of two 14-3-3 proteins, two FD-like proteins and two Hd3a proteins, designated as the florigen activation complex (FAC) [50]. This complex has been shown to bind the *OsMADS15* promoter (a homologue of *VRN1*) and induce flowering. Different combinations of FT-like, FD-like and 14-3-3 proteins can theoretically form a large number of possible different FACs, each varying in their constituent components. This system may provide flexibility to translate signals carried by FT*-*like mobile protein into tissue and developmental stage specific responses by modulating the expression of FD-like or 14-3-3 proteins interacting proteins in those tissues or developmental stages [51]. Our understanding of these complex interactions is in its infancy, but the results presented here suggest that changes in FT1 are associated with changes in the expression of other *FT-*like genes, which may help to coordinate the complex responses required during the transition from the vegetative to the reproductive phase. It would be interesting to test if the alteration of the expression of other *FT*-like genes affects the expression of *FT1*, or if this is a unidirectional hierarchical regulatory system in which *FT1* initiates a cascade of changes that affects the expression of the other members of the *FT-*like family.

### TILLING Mutants of *FT-A1* and *FT-B1* Homoeologs Provide New Variability to Engineer Wheat Flowering Time

The *ft-A1* mutant has a premature stop codon that eliminates more than half of the encoded protein whereas the *ft-B1*_263_ has an amino acid substitution within the conserved PEBP domain. Therefore, we initially expected the *ft-A1* mutant to exhibit a larger effect on flowering time than the *ft-B1*_263_ mutant. However, we observed the opposite; a significantly larger delay in flowering was associated with the *ft-B1*_263_ mutants than with the *ft-A1* mutant. This result suggests that the *FT-B1* homoeolog has a more important role in the regulation of flowering in tetraploid wheat than the *FT-A1* homoeolog.

The FT-A1 and FT-B1 proteins are identical, so differences in their transport efficiency or in their ability to induce flowering are unlikely to contribute to the observed differences in heading time. By contrast, the higher transcript levels of *FT-B1* relative to *FT-A1*, correlates well with the larger effect of *FT-B1* relative to *FT-A1* on wheat heading time, providing a simple explanation for the observed phenotypic effects. Similar differences have been described for hexaploid wheat, where *FT-B1* exhibits much higher expression levels than either the A or D homoeologs [15]. Although the *ft-A1* mutation showed no effect on flowering time, it was associated with a delay in heading time of 6.7 days in plants homozygous for the *ft-B1*_263_ mutation. This suggests that *FT-A1* is a hypomorphic allele, but that it still retains some ability to induce flowering under LD.

The slight differences in heading time between the two different *ft-B1*mutants and among the different combination of *ft-A1* and *ft-B1* alleles suggest that different combinations of *FT1* homoeologs (both mutant and natural alleles) can be used to precisely regulate heading time in wheat. This will become more important as wheat breeders try to adjust heading time of current wheat varieties in environments affected by global climate change.
